# Excess mortality and long-term disability from healthcare-associated carbapenem-resistant *Acinetobacter baumannii* infections: A nationwide population-based matched cohort study

**DOI:** 10.1371/journal.pone.0291059

**Published:** 2023-09-11

**Authors:** Chiu-Hsia Su, Li-Jung Chien, Chi-Tai Fang, Shan-Chwen Chang

**Affiliations:** 1 Division of Infection Control and Biosafety, Taiwan Centers for Disease Control, Taipei, Taiwan; 2 Institute of Epidemiology and Preventive Medicine, College of Public Health, National Taiwan University, Taipei, Taiwan; 3 Division of Infectious Diseases, Department of Internal Medicine, National Taiwan University Hospital, Taipei, Taiwan; 4 National Taiwan University College of Medicine, Taipei, Taiwan; Shiraz University of Medical Sciences, ISLAMIC REPUBLIC OF IRAN

## Abstract

**Background:**

Carbapenem resistance is perceived as a clinical challenge in the management of debilitated and immunocompromised patients who eventually will die from underlying diseases. We aimed to examine whether carbapenem resistance per se, rather than the underlying diseases, negatively affect outcomes, by comparing the excess mortality and morbidity from healthcare-associated infections (HAIs) caused by carbapenem-resistant *Acinetobacter baumannii* (CRAB) and carbapenem-susceptible *A*. *baumannii* (CSAB).

**Methods:**

This was a nationwide retrospective matched cohort study of hospitalized patients in 96 hospitals which participated in Taiwan Nosocomial Infection Surveillance (TNIS). A total of 2,213 patients with *A*. *baumannii* HAIs were individually matched to 4,426 patients without HAIs. Main outcomes were excess risks for one-year all-cause mortality and one-year new-onset chronic ventilator dependence or dialysis-dependent end-stage renal disease.

**Results:**

Excess one-year mortality was 27.2% in CRAB patients, compared with their matched uninfected inpatients, as well as 15.4% in CSAB patients (also compared with their matched uninfected inpatients), resulting in an attributable mortality of 11.8% (*P* <0.001) associated with carbapenem resistance. The excess risk associated with carbapenem resistance for new-onset chronic ventilator dependence was 5.2% (*P* <0.001). Carbapenem resistance was also associated with an extra cost of $2,511 per case of *A*. *baumannii* HAIs (*P* <0.001).

**Conclusion:**

Carbapenem resistance is associated with a significant disease burden in terms of excess mortality, long-term ventilator dependence, and medical cost. Further studies on effects of antimicrobial stewardship programs in decreasing this burden are warranted.

## Introduction

Healthcare-associated infections (HAIs), caused by the *Acinetobacter baumannii*, have emerged as a major global health problem posing significant threat to vulnerable hospitalized patients [1, 2]. This gram-negative bacterium is not only ubiquitous in nature but also capable of surviving for prolonged periods in healthcare environment [3, 4]. *A*. *baumannii* can causes pneumonia, bloodstream infections, urinary tract infection, and surgical site infections, with wide variations in the excess mortality rate estimates, from 7.8% to 34.0% in general patients [5, 6] and 2.6% to 43.0% in critically ill patients [7, 8], compared with patients without HAIs. Carbapenem antibiotics (e.g. imipenem and meropenem) were traditionally the most effective antimicrobials for treating *A*. *baumannii* infections [9]. Since 2000s, carbapenem resistance became a clinical therapeutic challenge [10]. Compared with carbapenem-susceptible *A*. *baumannii* (CSAB) HAIs, carbapenem-resistant *A*. *baumannii* (CRAB) HAIs were more likely to occur in debilitated or immunocompromised patients who eventually will die from underlying diseases [11]. Whether carbapenem resistance per se negatively affect the outcomes of *A*. *baumannii* HAIs remained uncertain. The question is important for assessing potential impact of antimicrobial stewardship programs that aim to reduce carbapenem resistance.

Existing literature includes five small matched cohort studies, which reported an increased short-term mortality by 1.3–6.9 folds in patients with CRAB HAIs compared to their matched patients with CSAB HAIs [10, 12–15]. Of the five studies, three studies focused on bloodstream *A*. *baumannii* infections (40–63matched-pairs) [10, 12, 15] and the other two studies examined all-type *A*. *baumannii* HAIs (42–91 matched-pairs) [13, 14]. None of these studies looked at long-term mortality or survivors’ functional status, such as chronic ventilator and dialysis dependence. Furthermore, the small sample size did not allow the researchers to control important confounding factors, particularly baseline patient characteristics.

A nationwide surveillance system, Taiwan Nosocomial Infection Surveillance (TNIS) collects HAI data throughout Taiwan. The TNIS data indicated that *A*. *baumannii* was responsible for 5.6% of all-type HAIs and 7.1% in bloodstream infections in Taiwan [16]. By 2021, up to 75.6% of those *A*. *baumannii* isolates in the intensive care units of medical centers were resistant to carbapenems [16]. This study aimed to investigate whether carbapenem resistance per se negatively affect the long-term outcomes of patients, including excess in mortality, new-onset chronic ventilator dependence, and new-onset dialysis-dependent end-stage renal disease, using the national health databases of the Taiwan Ministry of Health and Welfare.

## Methods

### Study design

This was a nationwide retrospective matched cohort study comparing the excess mortality and new-onset irreversible long-term morbidities (including ventilator dependence and dialysis-dependent end-stage renal disease) from HAIs caused by CRAB and that from HAIs caused by CSAB.

### Source of data

This work is part of the Taiwan Nosocomial Infection Surveillance (TNIS) Study [17]. To mitigate the potential confounding effects of the emergence of extensively drug-resistant *A*. *baumannii* (XDRAB) in Taiwan after 2008 [18] (TNIS data cannot differentiate between XARAB isolates and non-XDR CRAB isolates as tigecycline susceptibility was not routinely performed in most hospitals), this study exclusively included isolates collected from 2006 to 2008. The methodological details of TNIS Study, which applied matched cohort design to quantify excess mortality and long-term morbidity attributable to healthcare-associated infections, had been published [17]. S1 Appendix described the methods in cross-linking national health databases when applied to the present study in detail.

### Ethical statement

The study protocol was reviewed by the Research Ethic Committee of National Taiwan University Hospital (Taipei, Taiwan) and certified for exempt review in accordance with law and regulations (#201609005W), which does not require informed consent as all data were fully anonymized before we accessed them.

### Settings and HAI surveillance

The TNIS was established by Taiwan Centers for Diseases Control (Taipei, Taiwan). The participating hospitals used the United States Centers for Disease Control and Prevention (Atlanta, GA, USA) HAIs surveillance definitions [19].

### Statistical analysis

The excess risk of healthcare-associated *A*. *baumannii* HAIs was defined as the calculated the difference in mortality and new-onset organ failure risk between the *A*. *baumannii* HAI group and the uninfected group. The impact of carbapenem resistance was estimated by subtracting the excess mortality and morbidity risk, length of stay, and hospital cost of CRAB group from that of CSAB. All statistical analyses were performed using SAS, ver 9.2 (SAS Institute Inc., Cary, NC, USA). All P values were two-sided. Statistical significance was set at P < 0.05. The level of statistical significance for P values under multiple comparisons was set using Bonferroni’s correction.

## Results

**Fig 1:**
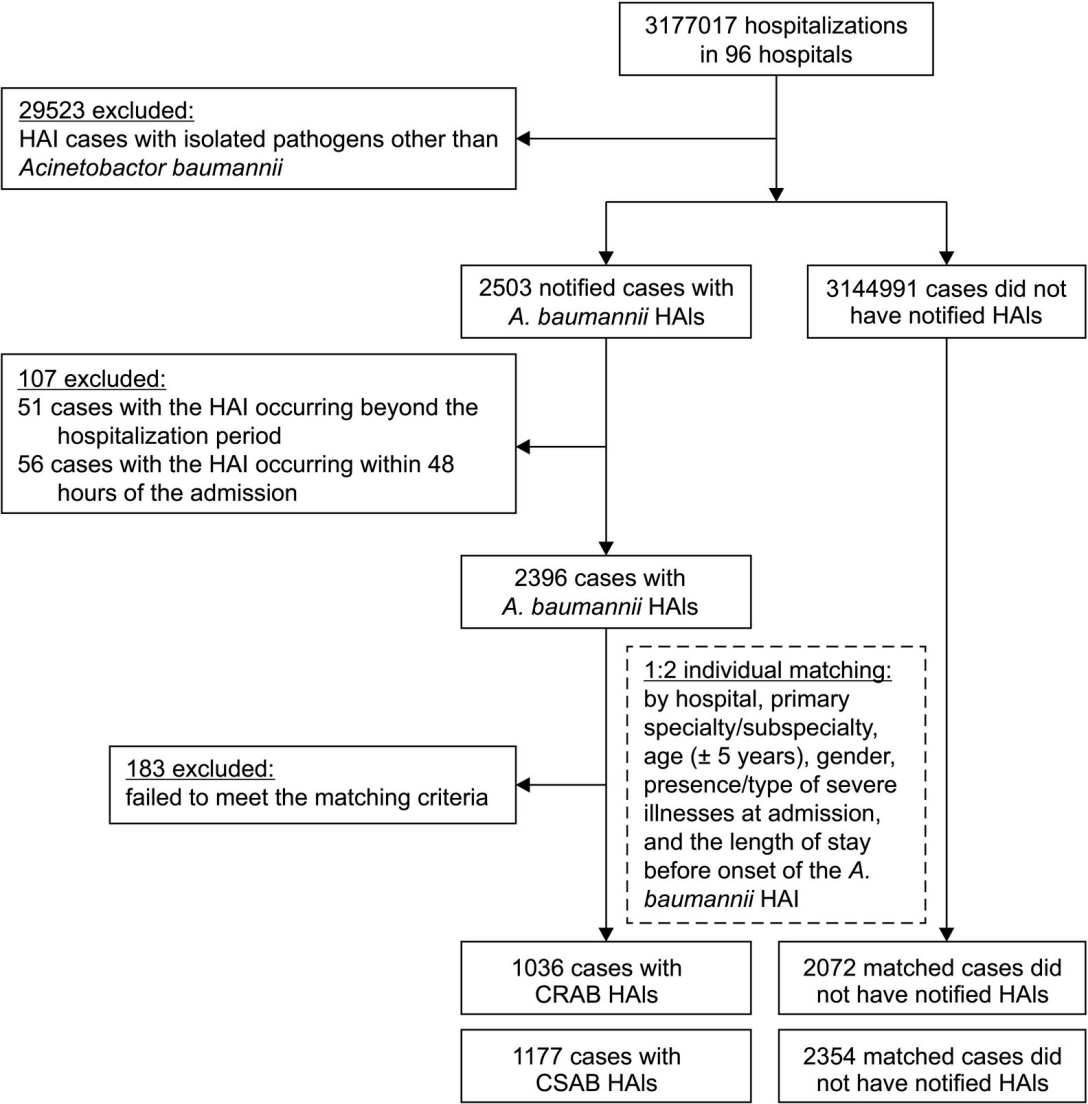
Enrollment of study subjects. Flow chart showed study design and patient selection for matching. Note: HAI, healthcare-associated infection; CRAB, carbapenem-resistant *Acinetobacter baumannii*; CSAB, carbapenem-susceptible *A*. *baumannii*.

Of the 32,026 HAI patients in 96 TNIS participating hospitals during the TNIS Study from 2006 to 2008, 7.8% (2,503/32,026) of isolated pathogen was *A*. *baumannii*, of which 95.7% (2,396/2,503) met the study inclusion criteria. Of them, 92.4% (2,213/2,396) were successfully matched to patients without HAIs (Fig 1). The *A*. *baumannii* HAI patients with unsuccessful matching (n = 183) had a significantly higher proportion of severe illness at baseline (on the date of admission) (13.7% vs. 2.8% for dialysis-dependent end-stage renal disease, *P* <0.001) and longer average length of stay before onset of the *A*. *baumannii* HAIs (31.7 days vs.17.1 days, *P* <0.001), than *A*. *baumannii* HAI patients with successful matching. The subsequent data analysis excluded the 183 patients who were unsuccessfully matched.

Comparison of baseline characteristics between 2,213 patients with *A*. *baumannii* HAIs and 4,426 patients without HAIs are shown in Table 1. The matched uninfected patients had a significantly higher proportion of ischemic heart disease (7.2% vs. 4.5%, *P* <0.001), diabetes mellitus (21.5% vs. 18.0%, *P* = 0.001), hypertension (23.7% vs. 13.6%, *P* <0.001), and lower average number of diagnoses (4.3 vs. 4.7, *P* <0.001), than *A*. *baumannii* HAI patients. Except the only 3 variables with significant differences, the remaining matching variables and validation variables had no significant differences between the two groups.

**Table 1:** Baseline characteristics of 2,213 matched pairs.

	*A*.*baumannii* HAI Patients (n = 2,213)	Matched Patients without HAIs (n = 4,426)	*P*-value
**Matching Variables**			
Age, mean±SD/median (IQR)	69±17/74 (59–81)	69±17/73 (59–81)	0.65
Sex, female (%)	656 (29.6)	1,312 (29.6)	1.0
Type of hospital, n (%)			
Medical center	696 (31.5)	1,392 (31.5)	1.0
Regional hospital	1,164 (52.6)	2,328 (52.6)	1.0
Local hospital	353 (16.0)	706 (16.0)	1.0
Primary specialty, ^a^ n (%)			
Neurosurgery	221 (10.0)	442 (10.0)	1.0
medicine	200 (9.0)	400 (9.0)	1.0
surgery	163 (7.4)	326 (7.4)	1.0
Neurology	103 (4.7)	206 (4.7)	1.0
Orthopedics	53 (2.4)	106 (2.4)	1.0
Plastic Surgery	51 (2.3)	102 (2.3)	1.0
Family Medicine	28 (1.3)	56 (1.3)	1.0
Rehabilitation Medicine	27 (1.2)	54 (1.2)	1.0
Severe illness, n (%)			
Cancer	364 (16.4)	728 (16.4)	1.0
End-stage renal disease	61 (2.8)	122 (2.8)	1.0
Liver cirrhosis with complications	33 (1.5)	66 (1.5)	1.0
Chronic ventilator dependence	50 (2.3)	100 (2.3)	1.0
Generalized autoimmune syndrome	19 (0.9)	38 (0.9)	1.0
Spinal injury or myeleterosis	3 (0.1)	6 (0.1)	1.0
Major trauma	12 (0.5)	24 (0.5)	1.0
**Validation Variables**			
Diagnosis, n (%)			
Ischemic heart disease	100 (4.5)	318 (7.2)	<0.001^b^
Congestive heart failure	138 (6.2)	279 (6.3)	0.91
Stroke	349 (15.8)	609 (13.8)	0.03
Diabetes mellitus	398 (18.0)	951 (21.5)	0.001^b^
Hypertension	302 (13.6)	1,050 (23.7)	<0.001^b^
Procedure, n (%)			
Total joint replacement	10 (0.5)	23 (0.5)	0.71
Coronary artery bypass graft	18 (0.8)	37 (0.8)	0.92
Laparoscopy	6 (0.3)	13 (0.3)	0.87
Medication, n (%)			
Antigout preparations	162 (7.3)	334 (7.5)	0.74
Antineoplastic agents	116 (5.2)	251 (5.7)	0.47
Statins	81 (3.7)	204 (4.6)	0.07
Streptokinase	14 (0.6)	16 (0.4)	0.12

^a^ Eight out of 15 primary specialties with the most patients were listed.

^b^ Statistically significant using Bonferroni’s correction (*P*<0.05/32 = 0.0016).

Abbreviations: HAI, healthcare-associated infection; SD, standard deviation; IQR, interquartile range.

**Fig 2:**
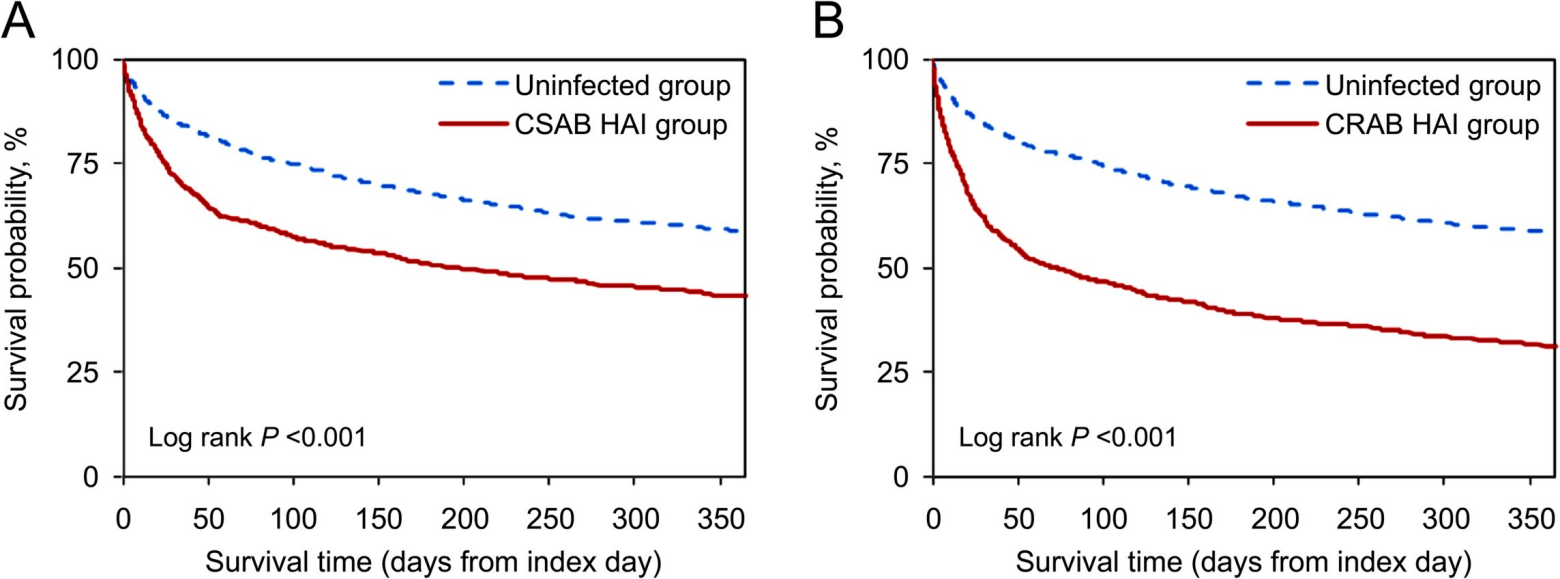
Kaplan-Meier survival curves. (A) CSAB patients (n = 1,177) and their matched uninfected patients (n = 2,354). (B) CRAB patients (n = 1,036) and their matched uninfected patients (n = 2,072). Note: CSAB HAI, carbapenem-susceptible *Acinetobacter baumannii* healthcare-associated infection; CRAB HAI, carbapenem-resistant *A*. *baumannii* healthcare-associated infection.

Patients with *A*. *baumannii* HAIs had an attributable in-hospital mortality, mortality within 30 days after discharge, and one-year mortality of 21.6%, 23.2%, and of 20.9%, respectively, compared with the matched uninfected patients. The excess risk of new-onset chronic ventilator dependence during hospitalization, within 30 days after discharge, and within one-year was 8.6%, 10.6%, and 10.2%, respectively. The excess mortality and excess risk of new-onset chronic ventilator dependence was highly statistically significant after Bonferroni correction for multiple comparisons and adjusting for the presence of ischemic heart disease, diabetes mellitus, and hypertension (all *P*s <0.001) (Table 2). The excess one-year mortality by site of infection was highest for nosocomial pneumonia (28.7%), followed by surgical site infections (21.3%), and bloodstream infection (17.9%). The excess one-year mortality by the type of antimicrobial resistance was 27.2% for CRAB and 15.4% for CSAB (all *P*s <0.001) (Table 3 and Fig 2). The excess hospital stay was 9.9 days and extra hospital cost was $6,096. The excess hospital stay and extra hospital cost were also significant in the subgroup analysis by site of infection, the type of antimicrobial resistance, and the presence of severe illnesses at admission (all *P*s <0.001) (Table 4).

**Table 2:** Excess risks for mortality and new-onset organ failure in patients with *A*. *baumannii* HAIs.

Outcomes	Endpoint of Observation^a^	*A*. *baumannii* HAI Patients	Matched Patients without HAIs	Excess Risk (%)	OR	Adjusted OR^‡^^c^
Mortality	Number at risk ^b^	2,213	4,426			
	Discharge	731 (33.0)	508 (11.5)	21.6	4.6 ^d^	4.4 ^d^
	30-day after discharge	909 (41.1)	790 (17.8)	23.2	3.9 ^d^	3.8 ^d^
	one-year	1,377 (62.2)	1,827 (41.3)	20.9	3.1 ^d^	3.1 ^d^
Chronic ventilator dependence	Number at risk ^b^	2,163	4,326			
	Discharge	250 (11.6)	129 (3.0)	8.6	5.4 ^d^	5.4 ^d^
	30-day after discharge	312 (14.4)	167 (3.9)	10.6	5.2 ^d^	5.2 ^d^
	one-year	373 (17.2)	303 (7.0)	10.2	3.3 ^d^	3.3 ^d^
Dialysis-dependent end-stage renal disease	Number at risk ^b^	2,152	4,304			
	Discharge	17 (0.8)	18 (0.4)	0.4	2.0	2.5
	30-day after discharge	23 (1.1)	30 (0.7)	0.4	1.6	2.0
	one-year	32 (1.5)	55 (1.3)	0.2	1.2	1.5

^a^ Follow-up duration from index date to endpoint of observation.

^b^ Number at risk: the number of patients who have not yet developed the outcomes at admission.

^c^ Adjusted for ischemic heart disease, diabetes mellitus and hypertension.

^d^ Statistically significant using Bonferroni’s correction (*P*<0.05/18 = 0.0028).

Abbreviations: HAI, healthcare-associated infection; OR, odds ratio.

**Table 3:** Subgroup analysis of excess one-year mortality.

	*A*. *baumannii* HAI Patients		Matched Patients without HAIs		% Difference	*P*-value
Variables	n	Event (%)	n	Event (%)		
One-year mortality, n (%)	2,213	1,377 (62.2)	4,426	1,827 (41.3)	20.9	<0.001^b^
By site of infection of index *A*. *baumannii* HAI patients						
Pneumonia	848	603 (71.1)	1,696	720 (42.5)	28.7	<0.001^b^
Bloodstream infection	584	363 (62.2)	1,168	517 (44.3)	17.9	<0.001^b^
Urinary tract infection	509	277 (54.4)	1,018	408 (40.1)	14.3	<0.001^b^
Surgical site infection	75	32 (42.7)	150	32 (21.3)	21.3	<0.001^b^
Others	197	102 (51.8)	394	150 (38.1)	13.7	<0.001^b^
By antimicrobial resistance of index *A*. *baumannii* HAI patients						
CSAB	1,177	666 (56.6)	2,354	969 (41.2)	15.4	<0.001^b^
CRAB	1,036	711 (68.6)	2,072	858 (41.4)	27.2	<0.001^b^
By presence of severe illnesses ^a^ at admission of index *A*. *baumannii* HAI patients						
No	1,676	976 (58.2)	3,352	1,156 (34.5)	23.7	<0.001^b^
Yes	537	401 (74.7)	1,074	671 (62.5)	12.2	<0.001^b^

^a^ Any of the 7 classes of severe illnesses (cancer, dialysis-dependent end stage renal disease, liver cirrhosis with complications, chronic ventilator dependence, generalized autoimmune syndrome, spinal injury/myeleterosis, and major trauma).

^b^ Statistically significant using Bonferroni’s correction (*P*<0.05/10 = 0.005).

Abbreviations: HAI, healthcare-associated infection; SD, standard deviation; CSAB, carbapenem-susceptible *A*. *baumannii*; CRAB, carbapenem- resistant *A*. *baumannii*.

**Table 4:** Subgroup analysis of excess hospital stay and medical costs.

	*A*. *baumannii* HAI Patients		Matched Patients without HAIs		Mean Difference	*P*-value
Variables	n	Mean (SD)	n	Mean (SD)		
Length of stay, mean (SD), days	2,213	35.5 (17.5)	4,426	25.6 (16.4)	9.9	<0.001^c^
By site of infection of index *A*. *baumannii* HAI patients						
Pneumonia	848	35.6 (16.6)	1,696	23.3 (15.2)	12.3	<0.001^c^
Bloodstream infection	584	31.9 (17.9)	1,168	24.5 (16.4)	7.4	<0.001^c^
Urinary tract infection	509	38.7 (17.3)	1,018	30.7 (17.3)	8.0	<0.001^c^
Surgical site infection	75	39.0 (18.4)	150	25.8 (17.1)	13.2	<0.001^c^
Others	197	35.9 (17.8)	394	25.6 (16.1)	10.3	<0.001^c^
By antimicrobial resistance of index *A*. *baumannii* HAI patients						
CSAB	1,177	33.7 (17.1)	2,354	24.1 (15.9)	9.6	<0.001^c^
CRAB	1,036	37.6 (17.6)	2,072	27.3 (16.8)	10.3	<0.001^c^
By presence of severe illnesses^*^ at admission of index *A*. *baumannii* HAI patients						
No	1,676	36.1 (17.6)	3,352	25.5 (16.4)	10.6	<0.001^c^
Yes	537	33.7 (16.9)	1,074	26.0 (16.5)	7.7	<0.001^c^
Cost of hospitalization, mean (SD), in US dollars ^b^	2,213	12,047 (8,581)	4,426	5,951 (6,009)	6,096	<0.001^c^
By site of infection of index *A*. *baumannii* HAI patients						
Pneumonia	848	12,567 (7,806)	1,696	5,260 (5,540)	7,306	<0.001^c^
Bloodstream infection	584	11,361 (9,090)	1,168	5,766 (5,842)	5,595	<0.001^c^
Urinary tract infection	509	11,017 (7,936)	1,018	7,235 (6,803)	3,782	<0.001^c^
Surgical site infection	75	14,830 (11,916)	150	6,627 (7,163)	8,204	<0.001^c^
Others	197	13,446 (9,733)	394	5,896 (5201)	7,549	<0.001^c^
By antimicrobial resistance of index *A*. *baumannii* HAI patients						
CSAB	1,177	10,324 (7,881)	2,354	5,404 (5,693)	4,921	<0.001^c^
CRAB	1,036	14,004 (8,921)	2,072	6,572 (6,293)	7,432	<0.001^c^
By presence of severe illnesses^a^ at admission of index *A*. *baumannii* HAI patients						
No	1,676	12,401 (8,800)	3,352	5,950 (5,990)	6,451	<0.001^c^
Yes	537	10,942 (7,760)	1,074	5,952 (6,071)	4,989	<0.001^c^

^a^ Any of the 7 classes of severe illnesses (cancer, dialysis-dependent end stage renal disease, liver cirrhosis with complications, chronic ventilator dependence, generalized autoimmune syndrome, spinal injury/myeleterosis, and major trauma).

^b^ At an exchange rate of 30 New Taiwan Dollars (NT$s) / US$.

^c^ Statistically significant using Bonferroni’s correction (*P*<0.05/20 = 0.0025).

Abbreviations: HAI, healthcare-associated infection; SD, standard deviation; CSAB, carbapenem-susceptible *A*. *baumannii*; CRAB, carbapenem- resistant *A*. *baumannii*.

Of the 2,213 *A*. *baumannii* HAI cases, the causal *A*. *baumannii* strains were CRAB in 1,036 cases (46.8%). Patients with CRAB HAIs (n = 1,036) had a longer length of stay in hospital before the onset of the *A*. *baumannii* HAI (18.8 days vs. 15.6 days), older age (70.2 years vs. 68.0 years), and higher proportion of occurring in intensive care units (54.7% vs. 34.2%), compared with patients with CSAB HAIs (n = 1,177) (all *P*s <0.001). These data implied the important differences in severity of underlying diseases between patients with CRAB HAIs and CSAB HAIs.

Table 5 summarizes the main outcomes of carbapenem resistance in *A*. *baumannii* HAIs. The excess one-year mortality was 27.2% among CRAB patients compared with their matched uninfected inpatients, and 15.4% among CSAB patients compared with their matched uninfected inpatients, resulting in an attributable mortality of 11.8% (all *P*s <0.001). The excess one-year mortality of cabapenem resistance was highest for bloodstream (27.6%), followed by urinary tract infections (12.4%), and pneumonia (4.4%). Carbapenem resistance had an excess risk for new-onset chronic ventilator dependence of 5.2% (*P*s <0.001) and had an extra hospital cost of $2,511 (*P* <0.001) (Table 6).

**Table 5:** Excess risks for mortality and new-onset chronic ventilator dependence attribute to carbapenem resistance.

	CRAB matched group				CSAB matched group				Attributable difference of excess risk
Outcomes	no. of pairs^b^	CRAB HAI Patients	Matched Patients without HAIs	Excess Risk	no. of pairs^b^	CSAB HAI Patients	Matched Patients without HAIs	Excess Risk	
Mortality, %									
Overall	1,036	68.6	41.4	27.2	1,177	56.6	41.2	15.4	11.8^a^
Pneumonia	449	73.9	43.2	30.7	399	67.9	41.6	26.3	4.4
Bloodstream infection	178	78.1	41.0	37.1	406	55.2	45.7	9.5	27.6^a^
Urinary tract infection	277	61.7	41.7	20.0	232	45.7	38.1	7.6	12.4^a^
Surgical site infection	31	38.7	19.4	19.3	44	45.5	22.7	22.8	-3.5
Other	101	56.4	40.1	16.3	96	46.9	25.5	21.4	-5.1
New-onset chronic ventilator dependence, %									
Overall	1,007	21.1	8.1	13.0	1,156	13.9	6.1	7.8	5.2^a^
Pneumonia	435	23.7	8.6	15.1	391	16.6	6.9	9.7	5.4^a^
Bloodstream infection	174	14.4	5.8	8.6	404	10.1	5.2	4.9	3.7
Urinary tract infection	268	22.4	10.3	12.1	223	17.9	6.3	11.6	0.5
Surgical site infection	30	10.0	3.0	7.0	44	11.4	5.7	5.7	1.3
Other	100	21.0	6.0	15.0	94	10.6	5.9	4.7	10.3^a^

^a^ Statistically significant using Bonferroni’s correction (*P*<0.05/12 = 0.0042).

^b^ Number at risk: the number of patients who have not yet developed the outcomes at admission.

Abbreviation: HAI, healthcare-associated infection; CRAB, carbapenem resistant *A*. *baumannii*; CSAB, carbapenem susceptible *A*. *baunammii*.

**Table 6:** Prolonged hospital length of stay and extra hospital cost attributable to carbapenem resistance.

	CRAB matched group				CSAB matched group				Attributable difference of mean difference
Outcomes	no. of pairs^c^	CRAB HAI Patients	Matched Patients without HAIs	Mean difference	no. of pairs^c^	CSAB HAI Patients	Matched Patients without HAIs	Mean difference	
Length of stay, days									
Overall	1,036	37.6	27.3	10.3	1,177	33.7	24.1	9.6	0.7
Pneumonia	449	36.4	23.8	12.6	399	34.8	22.7	12.1	0.5
Bloodstream infection	178	33.9	27.0	6.9	406	31.1	23.4	7.7	-0.8
Urinary tract infection	277	40.7	32.7	8.0	232	36.4	28.4	8.1	-0.1
Surgical site infection	31	47.6	29.5	18.1	44	33.0	23.2	9.8	8.3
Other	101	38.5	28.0	10.5	96	33.3	23.0	10.3	0.2
Cost of hospitalization, in US dollars^a^									
Overall	1,036	14,004	6,572	7,432	1,177	10,324	5,404	4,921	2,511^b^
Pneumonia	449	13,748	5,666	8,082	399	11,237	4,804	6,433	1,649^b^
Bloodstream infection	178	15,294	6,680	8,614	406	9,637	5,365	4,272	4,342^b^
Urinary tract infection	277	12,360	7,824	4,536	232	9,414	6,532	2,882	1,654
Surgical site infection	31	20,059	7,950	12,109	44	11,147	5,694	5,453	6,656
Other	101	15,520	6,558	8,962	96	11,264	5,200	6,064	2,898

^a^ At an exchange rate of 30 New Taiwan Dollars (NT$s) / US$.

^b^ Statistically significant using Bonferroni’s correction (*P*<0.05/12 = 0.0042).

^c^ Number at risk: the number of patients who have not yet developed the outcomes at admission.

Abbreviation: HAI, healthcare-associated infection; CRAB, carbapenem resistant *A*. *baumannii*; CSAB, carbapenem susceptible *A*. *baunammii*.

## Discussion

Healthcare-associated *A*. *baummannii* infection is a significant threat to vulnerable hospitalized patients with severe outcome and deaths. To the best of our knowledge, this study is the first nationwide population-based large cohort study to investigate the negative impact of carbapenem resistance on the outcomes of patients with *A*. *baumannii* HAIs. Our study included 1,177 CSAB and 1,036 CRAB patients to demonstrate the burden of carbapenem resistance. We showed that carbapenem resistance in patients with *A*. *baumannii* HAIs increased the risks for long-term mortality of 11.8%, disability (new-onset chronic ventilator dependence) of 5.2% and extra hospital cost of $2,511. Apart from nationwide cohort study, our study had explored the clinical long-term outcomes, adjusted the confounding factors by using individual matching to enhance comparability between CRAB and CSAB HAI patients. The testing results of the matching variables and the comparability-validation variables did show no significance between-group difference in most baseline characteristics.

Our study is consistent with the previous studies on the excess mortality associated with carbapenem resistance in *A*.*baumannii* HAI patients [10, 12–15]. Existing literature revealed that an excess mortality ranged from 1.6–30.0% for carbapenem resistance in *A*. *baumannii* HAI patients. The wide range of excess mortality is likely due to the smaller sample size and different types of infections [10, 12–15]. Furthermore, our findings validated the existing results and found an excess one-year mortality of 11.8% for carbapenem resistance in all-type *A*.*baumannii* HAI patients, in which bloodstream infections and urinary tract infections suffered the largest excess one-year mortality of 27.6% and 12.4% for carbapenem resistance, respectively. A bloodstream infection represents a systemic dissemination of the infection rather than a localized infection such as pneumonia, urinary tract infection, and surgical site infection. The limited availability of effective treatment options for carbapenem resistance in *A*.*baumannii* bloodstream infections can increase the risk of treatment failure and result in severe sepsis or septic shock, both of which were associated with a higher excess mortality [1, 20].

Apart from excess long-term mortality, our study also revealed that carbapenem resistance in *A*. *baumannii* HAI patients had an excess risk of long-term morbidity of new-onset chronic ventilator dependence. *A*. *baumannii* infections can cause severe outcome of acute organ dysfunction [21–23]. The pathogen frequently causes respiratory infections in mechanically ventilated patients and thus worsen the respiratory function after *A*. *baumannii* HAIs [24, 25]. In the present study, the most common *A*. *baumannii* HAIs was pneumonia, represented 38% of all-type *A*. *baumannii* HAIs. Our findings provided the evidence on irreversibility of CRAB HAI-associated ventilator dependence and showed the carbapenem resistance increased the risks for new-onset chronic ventilator dependence by 5.2% after *A*. *baumannii* HAIs.

The attributable risks of long-term mortality and morbidity emphasized the importance of hospital infection control for CRAB. The major risk factors of CRAB acquisition included prior exposure to antibiotics (especially carbapenems), longer hospital stay, invasive procedures, and admission to a ward with CRAB colonization pressure were documented as the risk factors [26–29]. A nationwide study demonstrated a strong positive association between hospital carbapenem consumption and CRAB prevalence, thus suggested that prudent use of carbapenems antibiotics is essential to control the spread of CRAB [30]. Preventing and controlling of multidrug-resistant organisms (MDROs) are not only a national priority but also are assumed responsibility for all hospitals [1, 31]. Otherwise, the burden of antimicrobial resistance will result in increased morbidity, mortality, and costs of health care. Our results indicated that controlling the spread of CRAB within hospitals may translate to a decrease in incidence of irreversible severe outcomes and substantial savings in the National Health Insurance medical expenditure, particularly when the cost of long-term respiratory care for those who depend on ventilator is considered.

This study presents limitations. First, our data were retrieved from the TNIS Study data which did not cover every hospital in Taiwan. However, we minimized the potential bias on estimating excess risk by individually matching the *A*. *baumannii* HAI patients to uninfected patients in the same hospital. Second, it is possible that part of the 4,426 matched uninfected patients might indeed have HAIs due to voluntary nature of TNIS participation, which would have caused the underestimation of excess risks for long-term mortality and morbidity. Third, the diversity in antibiotic treatment precluded meaningful comparison between different treatments in outcomes of CRAB. Fourth, this study exclusively included isolates collected from 2006 to 2008 to minimize potential bias resulting from treatment given for XDRAB infections after 2008. As a result, antibiotic treatment and patients’ outcomes would not confound by XDRAB infections. Fifth, underlying disease might play an important role in the outcomes of these patients. There remains a possibility of residual confounding in our study despite conducting a matched cohort study design and adjusting for presence of underlying diseases.

In conclusion, carbapenem resistance in patients with *A*. *baumannii* HAIs has significant negative effect on the long-term clinical outcomes on vulnerable hospitalized patients. Future study on the performance of antimicrobial stewardship program on the patients’ outcome should be take into consideration the long-term negative outcomes.

## Supporting information

S1 Appendix A detail description of study methods in this article. A detailed description of the TNIS Study methods involves study design, settings, patients with *A*. *baumannii* HAIs, matched patients without HAIs, validation of comparability, ascertainment of outcomes, and statistical analysis.
(DOCX)

S1 Checklist STROBE statement—checklist of items that should be included in reports of observational studies. (DOCX)
