# Supplementary material for: Excess mortality and long-term disability from healthcare-associated carbapenem-resistant Acinetobacter baumannii infections: A nationwide population-based matched cohort study
Source: PLoS One. 2023 Sep 11;18(9):e0291059. doi: 10.1371/journal.pone.0291059 (PMC10495011; doi:10.1371/journal.pone.0291059)
Supplement: S1 Appendix — A detailed description of the TNIS Study methods involves study design, settings, patients with A. baumannii HAIs, matched patients without HAIs, validation of comparability, ascertainment of outcomes, and statistical analysis. (DOCX) [file pone.0291059.s001.docx]

**S1 Appendix**

**Methodology of the TNIS Study**

**Study Design**

The Taiwan Nosocomial Infections Surveillance (TNIS) Study is a nationwide population-based, matched cohort study that aims to estimate risk of excess mortality and morbidity attributable to healthcare-associated infection using the national health databases of the Taiwan Ministry of Health and Welfare. The matching variables included patient age, sex, hospital, specialty, underlying diseases, and the length of stay before the onset of the HAIs caused by *Acinetobacter baumannii*. Main outcomes were excess risks of one-year all-cause mortality and one-year new-onset chronic ventilator dependence or dialysis-dependent end-stage renal disease.

**Settings**

The TNIS system of Taiwan Centers for Disease Control (CDC) started to collect healthcare-associated infection data in 2006. By 2008, 114 out of the total 495 hospitals in Taiwan had participated in the nationwide surveillance system and reported their HAIs patients. There were 114 hospitals, including 8 medical centers, 43 regional hospitals, and 63 local hospitals, with a total of 3,307,878 admissions covered by the Taiwan National Health Insurance (NHI) during 2006 to 2008. Taiwan CDC continued to enhance the platform of TNIS system and renamed the system to Taiwan Healthcare-associated infection and Antimicrobial resistance Surveillance System (THAS) in 2020.

**Patients with *A. baumannii* HAIs**

The reported HAI data in THAS included the patient’s age, sex, onset date of HAIs, site of infection, isolated pathogen, and antimicrobial susceptibility. The onset date of HAI was the date when the first clinical symptom(s)/sign(s) occurred, or the earliest positive culture was sampled. For linkage with the NHI databases, we included all reported *A. baumannii* HAIs patients that occurred at least 48 hours after admission during 2006 to 2008. If a patient was multiple episodes of HAIs during the hospitalization, only the first episode and its first isolated pathogen were considered. Patients with the HAI occurring within 48 hours of the admission or beyond the hospitalization period were excluded.

**Matched patients without HAIs**

Each *A. baumannii* HAI patient was individually matched (1:2 ratio) to patients without HAIs that were hospitalized in the same hospital during the same study period. The matching variables included age (within a 5-year difference), sex, hospital, primary specialty/subspecialty, and indicators of underlying disease severity (including the length of stay before onset of the *A. baumannii* HAI and the presence and type of severe illnesses at admission, such as cancer, dialysis-dependent end stage renal disease, liver cirrhosis with complications, chronic ventilator dependence, major trauma, generalized autoimmune syndrome, and spinal injury/myeleterosis). If there were more than two candidate uninfected patients, the two that had the closest admission dates to that of the *A. baumannii* HAI patient were chosen. If no suitable matched uninfected patient was found, the matching variable of subspecialty was reduced to just primary specialty. If a suitable matched uninfected patient still could not be identified, the matching process deemed to have failed.

The NHI databases to obtain patient data for matching and validation of comparability were used. In Taiwan, The NHI has a coverage rate of 99% because of universal health insurance. The NHI claims data recorded five major diagnoses (such as one primary diagnosis and up to four secondary diagnoses) for the patient, which were reported by the hospital based on the ICD–9–CM coding system. We ascertained the presence and type of severe illnesses using the Catastrophic Illness Registry, which is a subset of the NHI databases. There are 30 major categories of catastrophic illnesses for which patient copayment can be exempted.

**Validation of Comparability**

To validate comparability between the *A. baumannii* HAI patients and matched uninfected patients on baseline characteristics at admission, we tested the between-group difference on clinical variables unrelated to HAIs, such as the presence of ischemic heart disease, congestive heart failure, stroke, diabetes, hypertension, elective surgical procedures, and medications for treating cardiovascular and/or neoplastic disorders.

**Ascertainment of Outcomes**

The data on survival status and date of death was obtained from the National Death Registry (from Ministry of Health and Welfare, Taiwan), which contains all the death certificates of Taiwanese citizens. The data on new-onset chronic ventilator dependence and dialysis-dependent end-stage renal disease was ascertained from the Catastrophic Illness Registry. The date of Catastrophic Illness Certificate application was used as the onset date of chronic ventilator dependence and dialysis-dependent end-stage renal disease. To distinguish old events that were already present at admission from new-onset events that occurred after the index date, we defined the index date for *A. baumannii* HAI patients as the onset date of the *A. baumannii* HAI; that for matched uninfected patients was the admission date plus the length of stay before onset of the *A. baumannii* HAI of the matched case. The data of hospital costs were obtained from the NHI databases.

**Statistical Analysis**

We compared the main outcomes between the *A. baumannii* HAI group and the uninfected group using multivariate conditional logistic regression stratified by matched pairs, with adjustment for the effects of diabetes mellitus and hypertension. We compared the length of hospital stay and the hospital cost between two groups using the random effect model. We compared the survival curves between the two groups using the Kaplan-Meier method and log-rank test.
