# Supplementary material for: Excess mortality and long-term disability from healthcare-associated carbapenem-resistant Acinetobacter baumannii infections: A nationwide population-based matched cohort study
Source: PLoS One. 2023 Sep 11;18(9):e0291059. doi: 10.1371/journal.pone.0291059 (PMC10495011; doi:10.1371/journal.pone.0291059)
Supplement: S1 Checklist — (DOCX) [file pone.0291059.s002.docx]

STROBE Statement—checklist of items that should be included in reports of observational studies

|  | Item No. | Recommendation | Page  No. | Relevant text from manuscript |
| --- | --- | --- | --- | --- |
| **Title and abstract** | 1 | (*a*) Indicate the study’s design with a commonly used term in the title or the abstract | 1 | A nationwide retrospective matched cohort study as stated in the Abstract on page 2. |
|  |  | (*b*) Provide in the abstract an informative and balanced summary of what was done and what was found | 2 | Provided in the Abstract on page 2. |
| Introduction | | | |  |
| Background/rationale | 2 | Explain the scientific background and rationale for the investigation being reported | 3-4 | Included in the Introduction section on page 3-4. |
| Objectives | 3 | State specific objectives, including any prespecified hypotheses | 4 | Included in the Introduction section on page 4. |
| Methods | | | |  |
| Study design | 4 | Present key elements of study design early in the paper | 5 | Included in the Methods section on page 5 and S1 Appendix. |
| Setting | 5 | Describe the setting, locations, and relevant dates, including periods of recruitment, exposure, follow-up, and data collection | 5 | Included in the Methods section on page 5 and S1 Appendix. |
| Participants | 6 | (*a*) *Cohort study*—Give the eligibility criteria, and the sources and methods of selection of participants. Describe methods of follow-up  *Case-control study*—Give the eligibility criteria, and the sources and methods of case ascertainment and control selection. Give the rationale for the choice of cases and controls  *Cross-sectional study*—Give the eligibility criteria, and the sources and methods of selection of participants |  | Not applicable. |
|  |  | (*b*) *Cohort study*—For matched studies, give matching criteria and number of exposed and unexposed  *Case-control study*—For matched studies, give matching criteria and the number of controls per case | 5 | Included in the Methods section on page 5 and S1 Appendix. |
| Variables | 7 | Clearly define all outcomes, exposures, predictors, potential confounders, and effect modifiers. Give diagnostic criteria, if applicable | 5 | Included in the Methods section on page 5 and S1 Appendix. |
| Data sources/ measurement | 8* | For each variable of interest, give sources of data and details of methods of assessment (measurement). Describe comparability of assessment methods if there is more than one group | 5 | Included in the Methods section on page 5 and S1 Appendix. |
| Bias | 9 | Describe any efforts to address potential sources of bias | 26 | Addressed in the limitation parts in the Discussion section on page 26 |
| Study size | 10 | Explain how the study size was arrived at | 5 | Included in the Methods section on page 5 and S1 Appendix. |

Continued on next page

| Quantitative variables | 11 | Explain how quantitative variables were handled in the analyses. If applicable, describe which groupings were chosen and why | 5-6 | Included in the Methods section on page 5-6. |
| --- | --- | --- | --- | --- |
| Statistical methods | 12 | (*a*) Describe all statistical methods, including those used to control for confounding | 6 | Included in the Methods section on page 6. |
|  |  | (*b*) Describe any methods used to examine subgroups and interactions | 5-6 | Included in the Methods section on page 5-6 and S1 Appendix. |
|  |  | (*c*) Explain how missing data were addressed | 5 | Included in the Methods section on page 5 and S1 Appendix. |
|  |  | (*d*) *Cohort study*—If applicable, explain how loss to follow-up was addressed  *Case-control study*—If applicable, explain how matching of cases and controls was addressed  *Cross-sectional study*—If applicable, describe analytical methods taking account of sampling strategy | 5 | Included in the Methods section on page 5 and S1 Appendix. |
|  |  | (*e*) Describe any sensitivity analyses |  | Not applicable. |
| Results | | | | |
| Participants | 13* | (a) Report numbers of individuals at each stage of study—eg numbers potentially eligible, examined for eligibility, confirmed eligible, included in the study, completing follow-up, and analysed | 7 | Included in the Results section on page 7. |
|  |  | (b) Give reasons for non-participation at each stage | 7 | Included in the Results section on page 7. |
|  |  | (c) Consider use of a flow diagram | 7 | Included in the Results section on page 7 and presented in Figure 1. |
| Descriptive data | 14* | (a) Give characteristics of study participants (eg demographic, clinical, social) and information on exposures and potential confounders | 7-11 | Included in the Results section on page 7-11 and summarized in Table 1. |
|  |  | (b) Indicate number of participants with missing data for each variable of interest | 7 | Included in the Results section on page 7 and presented in Figure 1. |
|  |  | (c) *Cohort study*—Summarise follow-up time (eg, average and total amount) | 12 | Included in the Results section on page 12 and presented in Figure 2. |
| Outcome data | 15* | *Cohort study*—Report numbers of outcome events or summary measures over time | 12-24 | Included in the Results section on page 12-24 and summarized in Table 2-6. |
|  |  | *Case-control study—*Report numbers in each exposure category, or summary measures of exposure |  | Not applicable. |
|  |  | *Cross-sectional study—*Report numbers of outcome events or summary measures |  |  |
| Main results | 16 | (*a*) Give unadjusted estimates and, if applicable, confounder-adjusted estimates and their precision (eg, 95% confidence interval). Make clear which confounders were adjusted for and why they were included | 12-24 | Included in the Results section on page 12-24 and summarized in Table 2-6. |
|  |  | (*b*) Report category boundaries when continuous variables were categorized | 7-24 | Included in the Results section on page 7-24 and summarized in Table 1-6. |
|  |  | (*c*) If relevant, consider translating estimates of relative risk into absolute risk for a meaningful time period | 12-24 | Included in the Results section on page 12-24 and summarized in Table 3-6. |

Continued on next page

| Other analyses | 17 | Report other analyses done—eg analyses of subgroups and interactions, and sensitivity analyses |  | Not applicable. |
| --- | --- | --- | --- | --- |
| Discussion | | | | |
| Key results | 18 | Summarise key results with reference to study objectives | 25 | Included in the Discussion section on page 25. |
| Limitations | 19 | Discuss limitations of the study, taking into account sources of potential bias or imprecision. Discuss both direction and magnitude of any potential bias | 26 | Included in the Discussion section on page 26. |
| Interpretation | 20 | Give a cautious overall interpretation of results considering objectives, limitations, multiplicity of analyses, results from similar studies, and other relevant evidence | 25-26 | Included in the Discussion section on page 25-26. |
| Generalisability | 21 | Discuss the generalisability (external validity) of the study results | 26 | Included in the Discussion section on page 26. |
| Other information | |  | | |
| Funding | 22 | Give the source of funding and the role of the funders for the present study and, if applicable, for the original study on which the present article is based | 27 | Provided in the text on page 27. |

*Give information separately for cases and controls in case-control studies and, if applicable, for exposed and unexposed groups in cohort and cross-sectional studies.

**Note:** An Explanation and Elaboration article discusses each checklist item and gives methodological background and published examples of transparent reporting. The STROBE checklist is best used in conjunction with this article (freely available on the Web sites of PLoS Medicine at http://www.plosmedicine.org/, Annals of Internal Medicine at http://www.annals.org/, and Epidemiology at http://www.epidem.com/). Information on the STROBE Initiative is available at www.strobe-statement.org.
